# Collagen Fibril Orientation Instructs Fibroblast Differentiation Via Cell Contractility

**DOI:** 10.1002/advs.202301353

**Published:** 2023-05-30

**Authors:** Jiranuwat Sapudom, Shaza Karaman, Brian Chesney Quartey, Walaa Kamal Eldin Mohamed, Nick Mahtani, Anna Garcia‐Sabaté, Jeremy Teo

**Affiliations:** ^1^ Laboratory for Immuno Bioengineering Research and Applications Division of Engineering New York University Abu Dhabi Abu Dhabi 129188 UAE; ^2^ School of Engineering Ecole Polytechnique Federale de Lausanne Lausanne 1015 Switzerland; ^3^ Department of Mechanical and Biomedical Engineering Tandon School of Engineering New York University New York 11201 USA

**Keywords:** collagen alignment, extracellular matrix, fibroblast differentiation, fibrotic tissue, mechanobiology, myofibroblasts

## Abstract

Collagen alignment is one of the key microarchitectural signatures of many pathological conditions, including scarring and fibrosis. Investigating how collagen alignment modulates cellular functions will pave the way for understanding tissue scarring and regeneration and new therapeutic strategies. However, current approaches for the fabrication of three‐dimensional (3D) aligned collagen matrices are low‐throughput and require special devices. To overcome these limitations, a simple approach to reconstitute homogeneous 3D collagen matrices with adjustable degree of fibril alignment using 3D printed inclined surfaces is developed. By characterizing the mechanical properties of reconstituted matrices, it is found that the elastic modulus of collagen matrices is enhanced with an increase in the alignment degree. The reconstituted matrices are used to study fibroblast behavior to reveal the progression of scar formation where a gradual enhancement of collagen alignment can be observed. It is found that matrices with aligned fibrils trigger fibroblast differentiation into myofibroblasts via cell contractility, while collagen stiffening through a crosslinker does not. The results suggest the impact of collagen fibril organization on the regulation of fibroblast differentiation. Overall, this approach to reconstitute 3D collagen matrices with fibril alignment opens opportunities for biomimetic pathological‐relevant tissue in vitro, which can be applied for other biomedical research.

## Graphical abstract

A new approach to reconstitute 3D collagen matrices with adjustable fibril alignment degree is developed. The aligned fibrils trigger fibroblast differentiation, impacting the regulation of fibroblast differentiation. This approach offers opportunities for creating biomimetic pathological‐relevant tissue in vitro, aiding the understanding of tissue scarring, regeneration, and new therapeutic strategies.

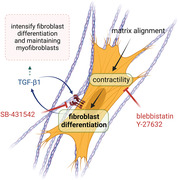

## 1 Introduction

Alignment of collagen fibrils is a hallmark of fibrotic tissues, which include the cancer microenvironment, obesity‐associated diseases, chronic inflammation, and scarring.^[^ ^1^, ^2^, ^3^ ^]^ It has been shown that the alignment of collagen fibrils provides changes not only in microarchitectural organization but also in the mechanical properties of the tissue.^[^ ^4^, ^5^ ^]^ During fibrosis, gradual changes in the mechanical properties of collagen can be observed due to alterations in its microstructure and organization, namely, collagen fibril thickening and alignment.^[^ ^6^, ^7^ ^]^ The change in the extracellular matrix (ECM) parameters might have a consequence for cells residing within; and thus, alter their functions and phenotypes. The major cells that are involved in fibrosis are fibroblasts and different subtypes of macrophages.^[^ ^8^, ^9^, ^10^ ^]^ The abundant cells found at fibrotic sites when compared to normal tissues are myofibroblasts. These differentiated fibroblasts are characterized by high expression of alpha‐smooth muscle actin (*α*SMA), excessive matrix component production, and a high contractile capability. The differentiation of fibroblasts into myofibroblasts is largely regulated by transforming growth factor beta‐1 (TGF‐*β*1);^[^ ^11^, ^12^ ^]^ however, the mechanical properties of the ECM can also trigger fibroblast differentiation via cell contractility.^[^ ^13^ ^]^ Recent works have shown that both fibroblasts and macrophages phenotypically change in response to mechanical and physical cues of their immediate surrounding microenvironment.^[^ ^14^, ^15^, ^16^ ^]^ A dynamic study on macrophage–fibroblast interactions in a coculture model demonstrated that macrophages preferentially migrate toward matrices actively deformed by fibroblasts and that microarchitectural changes can be sensed by macrophages several hundreds of micrometers away.^[^ ^17^ ^]^ Moreover, the percentage of attracted macrophages decreases with increasing collagen alignment. However, less is known about whether the gradual change in matrix organization alters the fate decision and cellular functions of fibroblasts, which will pave the way for understanding fibrotic progression and the development of therapeutic strategies for tissue fibrosis.

Due to the physiological and pathological relevance of collagen alignment, there have been many studies using various methods to mimic this aligned ECM microstructure to serve as biomimetic models of a range of tissues. For example, continuous cyclic stretching of a collagen sheet was used to rearrange collagen fibrils to a high degree of alignment, serving as a substrate for two‐dimensional (2D) culture studies.^[^ ^18^ ^]^ Other methods made use of microfluidic channels with controlled fluid flow allowing for shear forces^[^ ^19^, ^20^, ^21^ ^]^ or magnetic particles enmeshed in collagen hydrogel with an externally applied magnetic field^[^ ^4^, ^22^, ^23^ ^]^ to induce the alignment of collagen fibrils during the collagen fibrillation process. However, the success of achieving aligned collagen hydrogels through the latter approach has an average success rate of only 50%, and this is highly dependent on bead size and surface modification.^[^ ^23^ ^]^ These approaches also rely on the viscosity of the collagen solution; and thus, must be optimized for the desired concentration and batch‐to‐batch variations.^[^ ^24^ ^]^ It is worthwhile to note here that microfluidic‐based approach to aligning collagen fibrils in hydrogels can also achieve alignment control and in a gradient fashion if desired, with localized regions of shear‐induced inhomogeneity at the inlets and outlets whereby flow enters and leaves the system.^[^ ^19^, ^20^ ^]^ Another widely used method has to do with the use of three‐dimensional (3D) microfabrication techniques where natural or synthetic bioinks are printed in a shear‐induced aligned form.^[^ ^25^, ^26^, ^27^ ^]^ Despite the variety of methods developed for mimicking the alignment of collagen fibrils in vitro, these methods are invasive, low throughput, and may require specialized equipment and skill sets. The reconstitution of such matrices may not accurately mimic the in vivo ECM alignment, leaving foreign residuals (e.g., magnetic particles), and generally does not allow for adjusting the degree of fibril alignment.

In this study, we established a reproducible and well‐defined 3D collagen matrix that allows us to mimic the progression of tissue fibrosis where a gradual enhancement of collagen fibril alignment can be observed. We subsequently investigated the effects of collagen alignment of various degrees on human dermal fibroblasts. Cells were assessed at various resolutions using RNA sequencing, protein expression, and functional analysis. Our biomimetic model is robust and simple, high‐throughput, and most importantly, noninvasive; thus, leaving no residual component within the matrices, which can be applied to other biomedical applications.

## 2 Results and Discussion

Aligned collagen microarchitecture is known as a pathologically relevant extracellular matrix characteristic.^[^ ^1^, ^2^, ^3^ ^]^ Various methods have been devised to fabricate matrices with aligned collagen fibrils, namely, using microfluidic devices,^[^ ^19^, ^20^, ^21^ ^]^ paramagnetic microbeads,^[^ ^4^, ^22^, ^23^ ^]^ cyclic mechanical stretch devices,^[^ ^5^, ^18^, ^28^ ^]^ constrained cellular compaction,^[^ ^29^, ^30^ ^]^ and microextrusion 3D printing techniques.^[^ ^25^, ^26^, ^27^ ^]^ However, reported techniques are either inherently invasive and/or require special devices that essentially reduce reproducibility across research groups. In addition, they generally do not allow for high‐throughput reconstitution of homogeneous matrices, which allows for the study of downstream cell signaling. The comparison of various techniques for reconstituting 3D collagen matrices with fibril alignment is provided in Table S2, Supporting Information. We have overcome all these current limitations and further used the reconstituted matrices to reveal the progression of tissue fibrosis, where a gradual enhancement of collagen alignment can be observed, and also, its effects on fibroblast behavior as a result of these microarchitectural changes.

### 2.1 Reconstitution and Characterization of 3D Collagen Matrices With Adjustable Fibril Alignment Degree

**Figure 1:**
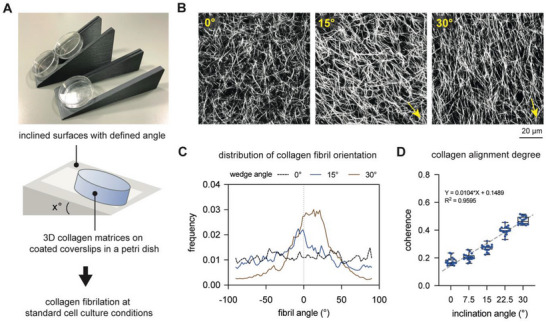
Reconstitution and characterization of 3D collagen matrices with adjustable fibril alignment degree. A) Schematic illustration of the developed approach to reconstitute collagen alignment using 3D‐printed inclined surfaces with defined angles. Collagen solution was transferred onto functionalized coverslips and placed onto the inclined surfaces prior to collagen fibrillation under standard cell culture conditions. B) Representative image of collagen matrices reconstituted on inclined surfaces with angles of 0°, 15°, and 30°. Yellow arrows represent the direction of alignment. C) Representative distribution plot of collagen fibril orientation of collagen matrices reconstituted on inclined surfaces with angles of 0°, 15°, and 30°. D) Quantitative analysis of the coherence index (CI) indicates the degree of collagen fibril alignment. The grey dashed line represents the linear fitting. For the quantification of CI, four different positions of each matrix condition were analyzed. Data are shown as box plots: box…median with 10th and 90th percentiles, error bars…minimum and maximum values. * indicates statistical significance of *p* < 0.05 using the Mann–Whitney test. Experiments were performed in four replicates.

To reconstitute 3D collagen matrices with aligned collagen fibrils, collagen solutions were prepared and transferred onto coated coverslips. During collagen fibrillation, coverslips were placed onto a planar surface (0°) or onto printed inclined surfaces with angles of 7.5°, 15°, 22.5°, and 30°, as depicted in **Figure** **1**A. The inclined surfaces have been designed to have a gap in the middle, allowing ample heat transfer for collagen fibrillation as the fibrillation temperature is known to affect collagen microstructure.^[^ ^31^ ^]^ It is important to note that there is non‐uniformity in the thickness of collagen matrices that are reconstituted on inclined surfaces with angles exceeding 40°. The mechanism of reconstitution of collagen alignment using our techniques relies on the sedimentation effects caused by gravitational forces acting on growing fibrils. As these fibrils continue to grow, they eventually become relatively large micron‐sized objects with sedimenting properties, which ultimately results in their movement in the direction of gravitation and subsequent alignment.

Upon collagen fibrillation, collagen matrices were fluorescently labeled by TAMRA‐SE and visualized using a confocal laser scanning microscope. As shown in Figure 1B, an increase in collagen alignment degree could be visually observed with an increase in the inclination angle. Representative images of collagen reconstituted on inclined surfaces with angles of 7° and 22.5° are depicted in Figure S1, Supporting Information. To show that the reconstituted collagen matrices from inclined surface with a 30° angle had uniform thickness (Figure S2, Supporting Information) and long‐range fibril alignment (Figure S3, Supporting Information), we imaged and quantified the collagen network using a low‐magnification objective. To further confirm the visual observation, we quantitatively analyzed the obtained images to gain insights into the distribution of collagen fibril orientation and their alignment degree. Analyzing collagen fibril orientation, collagen matrices fibrillated on inclined surfaces showed a Gaussian distribution, and the distribution peak was enhanced with increased inclination angle, while collagen matrices fibrillated on planar surfaces had uniformly distributed fibril orientation as fibrils were randomly oriented (Figure 1C). We further quantified the fiber alignment index by calculating the coherence index (CI) as it has been demonstrated to be an objective approach to quantify collagen alignment.^[^ ^32^ ^]^ CI ranges between 0 and 1, where 0 represents a perfectly random distribution of fibrils, while an index of 1 represents perfect fibril alignment. As shown in Figure 1D, the CI of collagen matrices fibrillated on planar surfaces was 0.18 ± 0.03, while collagen matrices fibrillated on inclined surfaces showed an increase in CI from 0.21 ± 0.03 and 0.47 ± 0.03 for inclination angles of 7.5° and 30°, respectively. The CI is proportional to the inclination angle, as shown by linear fitting (Figure 1D, gray dashed line, *R* ^2^ = 0.9595). It is important to note that the process of collagen fibrillation involves the self‐assembly of collagen monomers and the entanglement of fibrils to form a stable 3D network and cannot achieve the level of alignment degree that can be produced using bioprinting techniques. To ensure that the lower CI values are not biased by the analysis methods, phantom images of ideal and collagen fibril arrangements are created and analyzed regarding their CI (Figure S4, Supporting Information). Analysis of their CI revealed that increasing the number of aligned fibrils while keeping the number of random collagen fibrils constant resulted in a slight change in the CI value from 0.41 to 0.49 (Figure S4, Supporting Information), highlighting the low CI in our aligned collagen matrices.

Overall, using inclined surfaces with different angles allows us to mimic the gradual change in randomly organized collagen fibrils toward aligned collagen fibrils, mimicking the progression of tissue fibrosis. In addition, our CI analysis suggests that using our approach, the collagen alignment degree can be fine‐tuned to recapitulate human normal skin (CI: 0.21 ± 0.10), normotrophic skin (CI: 0.41 ± 0.13), hypertrophic skin (CI: 0.46 ± 0.16), and keloid scar skin (CI: 0.44 ± 0.15).^[^ ^33^ ^]^ Overall, our established approach using 3D printed inclined surfaces with different angles is able to reconstitute well‐defined and homogeneous collagen matrices with an adjustable fibril alignment degree, which is in the range of human normal skin tissue and scars.

**Figure 2:**
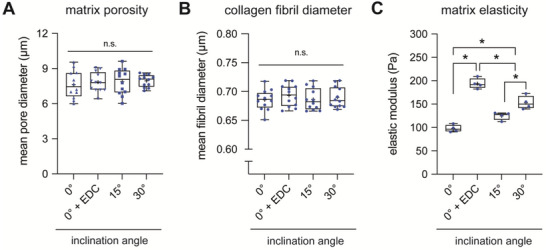
Topological and mechanical characterization of reconstituted 3D collagen matrices. Topological analysis of 3D collagen matrices for A) mean pore diameter and B) mean fibril diameter was performed using a custom‐made image analysis toolbox. Three different positions of each matrix condition from four different samples were analyzed. C) Bulk matrix elastic modulus was quantified using a non‐destructive contactless rheometer from four different samples of each matrix condition. Data are shown as box plots: box…median with 10th and 90th percentiles, error bars…minimum and maximum values. * indicates statistical significance of *p* < 0.05 using the Mann–Whitney test.

To determine whether the collagen alignment degree impacts fibroblast behavior, matrices reconstituted on a planar surface (0°) and inclined surfaces with 15° and 30° angles were chosen. Prior to doing that, we quantitatively characterized the topological and mechanical properties of these reconstituted matrices. Mean pore diameter and fibril diameter were analyzed using a custom‐made image analysis toolbox, and both parameters were found to be consistent for all matrices and reconstitution conditions (**Figure** **2**A,B). The bulk matrix elastic modulus was characterized using a non‐destructive contactless rheometer. We found an increase in the elastic modulus with increasing alignment degree from 97.80 ± 7.8 Pa to 124.7 ± 8.4 Pa and 153 ± 14.3 Pa for 0°, 15°, and 30° inclination angles, respectively (Figure 2C). Our data are in line with other studies demonstrating that aligned collagen matrices exhibited a higher elastic modulus than collagen matrices with a random fibril distribution.^[^ ^4^, ^34^ ^]^ It is known that matrix stiffness can modulate fibroblast functions.^[^ ^14^ ^]^ To decipher the impact of collagen alignment and matrix stiffness on fibroblast behavior, we enhanced the stiffness of collagen matrices reconstituted on a planar surface using a carbodiimide crosslinker (0° + EDC; E = 193.8 ± 11.3 Pa) while keeping the mean pore diameter (Figure 2A) and fibril diameter (Figure 2B) at levels similar to those of uncrosslinked and aligned matrices, as previously demonstrated.^[^ ^35^ ^]^ A representative image of EDC‐crosslinked matrices is shown in Figure S1, Supporting Information. We would like to highlight that; although collagen matrices are crosslinked with EDC, their elastic modulus is relatively low and falls within the range of hundreds of Pascals. This is lower than the elastic modulus of native tissues, which contains additional ECM components. Nevertheless, even a small increase in collagen matrix elasticity has been demonstrated to have an impact on cell behavior in various cell types.^[^ ^15^, ^36^, ^37^, ^38^ ^]^ In addition, anisotropy in microarchitecture of the collagen matrices will result in significantly differing bulk elastic properties when samples are measured longitudinal and perpendicular to collagen fibril alignment. It is expected that the longitudinal stiffness, parallel to the direction of alignment, will be considerably greater than perpendicular stiffness. Unfortunately, the current method used to measure the elastic modulus is unable to capture these differences. In our case, the measured modulus is to provide a comparison of elastic modulus between matrices.

### 2.2 Collagen Fibril Alignment Modulates the Orientation and Elongation of Fibroblasts

**Figure 3:**
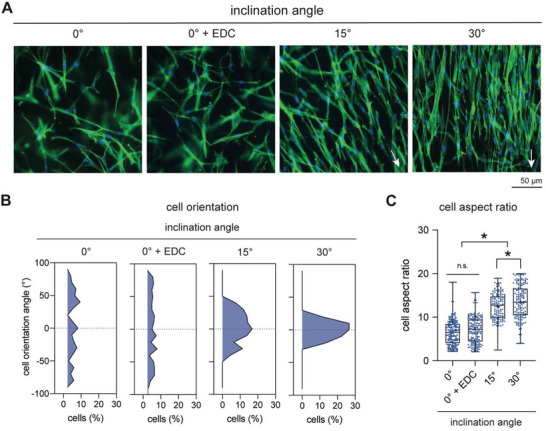
Cell orientation and elongation of fibroblasts in reconstituted 3D matrices with aligned and random fibrils. A) Representative images of fibroblasts cultured onto reconstituted 3D collagen matrices. White arrows represent the direction of alignment. B) Distribution plot of the orientation of the cell body in dependence on collagen fibril organization. C) Quantitative analysis of the cell aspect ratio as an indicator of cell elongation in dependence on collagen fibril organization. At least 200 cells were analyzed from four different positions and four matrices of each matrix condition. Data are shown as box plots: box…median with 10th and 90th percentiles, error bars…minimum and maximum values. * indicates statistical significance of *p* < 0.05 using the Mann–Whitney test.

It has been reported that cell orientation and elongation are altered by the organization of collagen fibrils.^[^ ^28^, ^39^ ^]^ To demonstrate this aspect, we cultured primary human dermal fibroblasts onto our well‐characterized 3D collagen matrices for 3 days. Afterward, the cell cytoskeleton and nucleus were stained and visualized using an epifluorescence microscope. As shown in **Figure** **3**A, fibroblasts cultured on collagen matrices reconstituted on a planar surface (0°) were randomly oriented. An increase in the matrix stiffness using an EDC crosslinker did not affect cell orientation. However, fibroblasts cultured onto collagen matrices reconstituted at 15° and 30° inclination angles were collectively aligned. In addition, we found an enhancement in stress fibers in fibroblasts cultured onto matrices with aligned collagen fibrils when compared to cells cultured on randomly organized fibrils of both uncrosslinked and crosslinked conditions. Quantitative analysis of the cell orientation confirmed our visual observation (Figure 3B). Interestingly, we observed that the orientation of cells cultured onto collagen matrices reconstituted on 15° collagen matrices appeared to be more dispersed when compared to the cells cultured onto the 30° counterpart. These data suggest that the orientation of the cell body is correlated with the collagen alignment degree. In addition to the analysis of cell orientation, we quantified the cell aspect ratio as an indication of cell elongation. As shown in Figure 3C, we found that cell elongation is modulated by the alignment degree of collagen fibrils. However, an increase in the bulk elastic modulus of the randomly distributed collagen fibrils (0° + EDC) does not affect cell elongation. Our data suggest that the organization of collagen fibrils, but not of matrix stiffness, modulates cell elongation in fibroblasts.

### 2.3 RNA Sequencing Reveals Collagen Alignment‐Mediated Changes in the Transcriptome of Fibroblasts Toward Myofibroblasts

**Figure 4:**
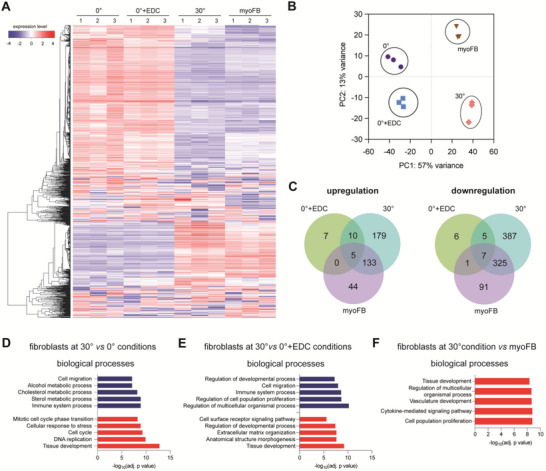
Transcriptome analysis using RNA sequencing of fibroblasts cultured onto matrices with randomly organized and aligned collagen fibrils. Functional transcriptome analysis was analyzed using the iDEP web‐based tool. A) Heat map of overall gene expression levels (blue‐low expression; red‐high expression). B) Principal component analysis of gene expression data. C) The Venn diagram shows up‐ and downregulated differentially expressed genes with an FDR cutoff of 0.05 and a minimal fold change of 2. Analysis of biological processes of cells cultivated under D) 30° versus 0° conditions, E) 30° versus 0° + EDC conditions, and F) 30° conditions versus myofibroblasts using the generally applicable gene‐set enrichment (GAGE) method. RNA sequencing was performed in three independent experiments.

As demonstrated above, fibroblasts adapted their cell orientation and spreading behavior in response to collagen fibril alignment (Figure 3). To reveal to what extent collagen fibril alignment affects fibroblast differentiation and functions, we performed RNA sequencing of fibroblasts cultured on 0°, 0° + EDC, 30°, and myofibroblasts (MyoFBs). In this case, fibroblasts were cultured onto a random matrix (0°) in the presence of TGF‐*β*1 to differentiate them into myofibroblasts and were used as a control. As shown in **Figure** **4**A, we found that the gene expression patterns of fibroblasts cultured on randomly distributed collagen fibrils (both 0° and 0° + EDC) were similar, while fibroblasts cultured onto an aligned fibril matrix (30°) demonstrated a transcriptome profile similar to that of myofibroblasts. Principal component analysis (PCA) confirmed that there is a clear difference between cells cultivated on random (0° and 0° + EDC) and aligned matrices (30°), as demonstrated by PC1 (principal component 1) shown in Figure 4B. PCA also revealed differences in the transcriptomes of fibroblasts on an aligned matrix (30°) and myofibroblasts. From the RNASeq data, we further analyzed differentially expressed genes (DEGs) with a false discovery rate (FDR) cut‐off of 0.05 and a minimal fold change of 2 for all conditions compared to cells cultured on a matrix with randomly distributed fibers without the EDC crosslinker (0°). As shown in Figure 4C, up‐ and down‐regulated DEGs were plotted as a Venn diagram. Fibroblasts cultivated on matrices with randomly distributed fibers (both 0° and 0° + EDC), irrespective of the bulk matrix stiffness/crosslinking, demonstrated less change in DEGs, confirming a similar transcriptome profile of both conditions. Fibroblasts cultivated on matrices with aligned fibrils (30°), however, showed an increase in both upregulated and downregulated DEGs when compared to cells cultivated on random matrices (both 0° and 0° + EDC), indicating that fibroblast function changes when cultivated on matrices with aligned fibrils. Interestingly, fibroblasts on an aligned matrix (30°) share common DEGs with myofibroblasts, but both cell types still exhibit distinct DEGs. To investigate enrichment in biological pathways, gene set analysis using the generally applicable gene‐set enrichment (GAGE) method was performed. This analysis allows us to unbiasedly predict the biological pathway independent of the identified DEGs as DEGs depend heavily on the criteria set for the cut‐off, in our case, an FDR cut‐off of 0.05 and a minimal fold change of 2. The GAGE analysis allowed us to overcome these issues by using pathway knowledge; therefore, small but coordinated changes in the gene set can be used to predict the up‐ or downregulation of specific biological pathways. By doing this, we first analyzed the enriched biological pathways of cells in matrices with random fibrils (0° and 0° + EDC); no significant enrichment in specific pathways was found, as also suggested by minimal numbers of DEGs (Figure 4C). By comparing fibroblasts cultured on matrices with aligned fibrils (30°) to (both 0° and 0° + EDC), we found a common downregulation in biological pathways associated with cell migration (Figure 4D,E). In addition, gene sets in tissue development pathways have been shown to be upregulated in aligned matrices when compared to cells cultivated on matrices with random fibrils (0° and 0° + EDC). It also appears that fibroblasts cultured in matrix with aligned fibrils (30°) exhibited higher gene sets associated with cell proliferation when compared to matrices with random fibrils (0°) and enhanced expression of gene sets associated with matrix organization when compared to crosslinked matrices with random fibrils (0° + EDC). All these gene sets indicated the differentiation of fibroblasts into myofibroblasts as they are nonmotile, contractile, and highly proliferative cells.^[^ ^40^, ^41^ ^]^ In addition, fibroblasts cultured on aligned matrix possessed a transcriptome profile similar to that of myofibroblasts. By comparing their biological pathways using GAGE, we found an enrichment in gene sets associated with tissue development, regulation of multicellular organismal processes, cytokine‐mediated signaling pathways, and cell population proliferation (Figure 4F), suggesting a higher cellular activity of fibroblasts in the aligned matrix.

In sum, the transcriptome analysis using RNA sequencing suggests that stiffening of the random matrix using a crosslinker minimally affected the transcriptome profile of fibroblasts, whereby matrix alignment appears to instruct fibroblast differentiation into myofibroblasts, as demonstrated by a similar transcriptome profile and enhanced expression of gene sets associated with myofibroblasts.

### 2.4 Aligned Matrices Instruct Fibroblast Differentiation but Modulate Distinct Cytokine Expression Profiles

**Figure 5:**
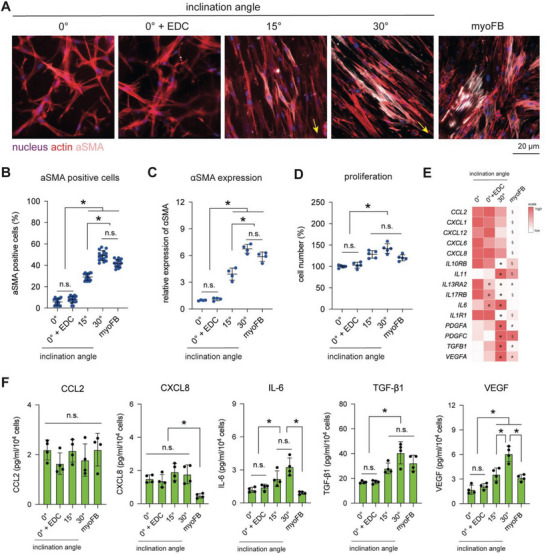
Fibroblast differentiation in matrices with randomly organized and aligned collagen fibrils. Fibroblasts were cultured onto reconstituted matrices for 3 days. Fibroblasts were analyzed regarding their differentiation into myofibroblasts, proliferative capacity, and cytokine secretion. Yellow arrows represent the direction of alignment. A) Representative images of fibroblasts stained with Hoechst‐33421 (nucleus; blue), phalloidin (actin; red), and *α*SMA (myofibroblast marker; grey) (scale bar = 20 µm). B) The number of *α*SMA‐positive cells was manually counted from the obtained images. At least four random positions per matrix were analyzed. C) The expression of *α*SMA was confirmed using RT‐qPCR. D) The number of cells was analyzed by counting cells using flow cytometry. Data were normalized to cell number from 0° conditions. Experiments were performed in four replicates. * indicates a significance level of *p* < 0.05 using the Mann–Whitney test. E) Expression of secreted cytokines analyzed from RNA‐Seq data. Data are presented as a heat map (red‐high expression; white‐low expression). Symbols, o (0° vs 0° + EDC), * (0° vs 30°), § (0° vs myoFB), and # (30° vs myoFB), indicate a significance level of *p* < 0.05 using the Mann–Whitney test. F) Secretion of CCL2, CXCL8, IL‐6, TGF‐β1 and VEGF quantified using bead‐based multiplex ELISA. * indicates a significant level of p < 0.05 using the Mann‐Whitney test.

From transcriptome analysis, we hypothesized that matrix alignment might trigger fibroblast differentiation, as clued by enhanced expression of gene sets in proliferation and matrix remodeling, which are incidentally reported characteristics and found in the myofibroblast transcriptome (Figure 4). To address this, we stained cells with anti‐*α*SMA antibody and manually quantified the obtained images regarding the number of *α*SMA‐positive cells. *α*SMA expression is a well‐known marker for myofibroblasts.^[^ ^42^ ^]^ **Figure** **5**A shows representative images of immunocytochemical staining of nuclei (blue), actin (red), and *α*SMA (gray). It could be visually observed that cells cultivated on aligned matrices, both 15° and 30°, presented *α*SMA expression similar to that of myofibroblasts, while it could not be observed in fibroblasts cultured on both random fibrils (0° and 0° + EDC). We further quantified the number of *α*SMA‐positive cells from the obtained images. As shown in Figure 5B, we found an increase in *α*SMA cells in aligned matrices (both 15° and 30°) when compared to random matrices (0° and 0°+EDC) independent of matrix stiffening. Quantitative image analysis confirmed the visual observation. In addition, the number of *α*SMA‐positive cells increased with increasing alignment degree. Cells cultured on highly aligned collagen (30°) appear to show a slight increase, albeit without significant difference, in *α*SMA‐positive cells when compared to myofibroblasts. Similarly, we observed an increase in *α*SMA gene expression as the inclination angle increased (Figure 5C). These data indicate that the degree of matrix alignment can modulate the level of fibroblast differentiation.

In addition to *α*SMA expression, cellular proliferation is another important feature of myofibroblasts, as reported elsewhere^[^ ^40^, ^41^ ^]^ and evident from our transcriptome analyses. We therefore analyzed the proliferation of cells by counting cells using flow cytometry. As shown in Figure 5D, we found enhanced cell proliferation in cells cultivated onto aligned matrices (both 15° and 30°) and myofibroblasts when compared to random matrices (both 0° and 0° + EDC). These data indicate that an increase in matrix stiffness in random matrices (0° + EDC vs 0°) does not affect cell proliferation in fibroblasts. In addition, it appears that fibroblasts cultivated onto an aligned matrix (30°) exhibited a slightly higher cell number than myofibroblasts, highlighting higher proliferative activity, as shown in the transcriptome analysis (Figure 4F). A slight increase, but not significant, in cell number could be observed with increasing alignment degree. Overall, along with *α*SMA expression, enhanced cell proliferation also supports our notion that matrix alignment instructs the differentiation of fibroblasts into myofibroblasts.

To further reveal functional differences of fibroblasts in random and aligned matrices to a greater extent, we analyzed the gene expression of cytokines based on our RNA sequencing data. As shown in Figure 5E, a heat map of the gene expression of major cytokines involved in cell–cell interactions was plotted. Random matrices with enhanced stiffness (0° + EDC vs 0°) significantly enhanced the gene expression of IL13RA2, IL17RB, and IL6. Aligned matrices significantly increased the expression of IL10RB, IL11, IL1R1, PDGFA, PDGFC, TGFB1, and VEGF in fibroblasts compared with random matrices (both 0° and 0° + EDC). Interestingly, myofibroblasts appear to express less CCL2, CXCL1, CXCL12, CXCL6, and CXCL8 than fibroblasts on both random (0° and 0° + EDC) and aligned matrices (30°).

We next confirmed the expression of specific cytokines, namely, CCL2, CXCL8, IL‐6, TGF‐*β*1, and VEGF, using multiplex bead‐based ELISA, as these cytokines are important for tissue fibrosis (Figure 5F). For all conditions, we found no change in CCL2, which is involved in monocyte recruitment. Myofibroblasts appeared to secrete less CXCL8 but maintained IL‐6 levels similar to those of fibroblasts in random matrices (both 0° and 0° EDC). CXCL8 regulated the directional migration of leukocytes and diminished wound contraction,^[^ ^43^ ^]^ while IL‐6 is known as a fibrotic mediator and triggered collagen production.^[^ ^44^ ^]^ Cells cultured on aligned matrices (both 15° and 30°) significantly increased their secretion of IL‐6, TGF‐*β*1, and VEGF. In addition, those cytokines increased with the degree of alignment when comparing cells on 15° with 30°, but the increment was significant only for VEGF. TGF‐*β*1 was involved in the differentiation of fibroblasts and maintenance of the myofibroblast phenotype^[^ ^12^, ^41^, ^45^ ^]^ and could enhance collagen production and matrix remodeling in combination with IL‐6.^[^ ^46^ ^]^ VEGF, on the other hand, was important for initiating and directing angiogenesis, which was involved in tissue fibrosis and had been proposed as a therapeutic target along with TGF‐*β*1 inhibitors to resolve fibrosis.^[^ ^47^ ^]^ As shown in Figure 5F, IL‐6, TGF‐*β*1 (not significant), and VEGF were secreted more by fibroblasts in aligned collagen (30°) when compared to myofibroblasts, which helps to explain the accumulation of myofibroblasts and excessive production of ECM components in fibrotic tissues.

Overall, our *α*SMA expression analyses, proliferation studies, and cytokine secretion studies data manifested and confirmed the results from RNA‐Seq that fibril organization within collagen matrices could instruct myofibroblast differentiation in dependence of alignment degree, which we also demonstrated using *α*SMA staining and gene expression, as well as enhanced cell proliferation. In addition, our data reveal that fibroblasts cultured onto aligned matrices show differential expression patterns and levels of cytokines compared to myofibroblasts activated via TGF‐*β*1. This result indicates that the organization of collagen fibrils can modulate distinct cellular functions and their cytokine secretion profiles that in turn mediate communication with other cells in the microenvironment.

### 2.5 Matrix Remodeling is Enhanced in the Aligned Matrix

**Figure 6:**
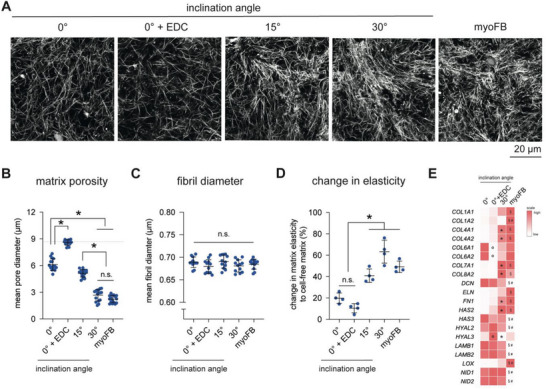
Matrix remodeling by fibroblasts in matrices with randomly organized and aligned fibrils. Fibroblasts were cultured onto reconstituted matrices for 3 days. Matrices were then decellularized and characterized regarding their topological and mechanical properties. A) Representative images of decellularized matrices (scale bar = 20 µm). Topological parameters, namely, B) matrix porosity and C) fibril diameter, were quantified using a custom‐built image analysis toolbox. D) The change in matrix elasticity was calculated by normalizing the obtained data to the matrix elasticity before cell seeding. Data are presented as a dot plot with mean and standard deviation. Experiments were performed in at least four replicates. * indicates a significance level of *p* < 0.05 using the Mann–Whitney test. E) Expression of matrix components analyzed from RNA‐Seq data. Data are presented as a heat map (red‐high expression; white‐low expression). The symbols o (0° vs 0° + EDC), * (0° vs 30°), § (0° vs myoFB), and # (30° vs myoFB) indicate a significance level of *p* < 0.05 using the Mann–Whitney test.

Another parameter that is an important measure of fibroblast function is the ability to perform matrix remodeling. To investigate this, we first decellularized matrices that housed fibroblasts for 3 days, visualized them using cLSM; and then, quantified the mean pore and fibril diameters using the custom‐built image analysis toolbox. Representative images of decellularized matrices under different conditions are shown in **Figure** **6**A. It could be visually observed that matrices of myofibroblasts and fibroblasts cultured on aligned matrices were strongly remodeled when compared to the collagen microarchitecture of random matrices. These data support that fibroblasts are triggered to differentiate in aligned collagen matrices as myofibroblasts have been reported to exhibit a contractile phenotype that is capable of matrix remodeling.^[^ ^9^, ^40^ ^]^ By analyzing the mean pore diameter, we found that matrices remodeled by myofibroblasts showed a significant reduction in pore diameter compared to that of fibroblasts in random matrices (0° and 0° EDC), as shown in Figure 6B. Our data also demonstrated that the crosslinked matrices were minimally remodeled by fibroblasts. For aligned matrices, we found that the reduction in pore diameter due to remodeling appeared to be dependent on the alignment degree and reached a pore diameter range similar to that of the matrix remodeled by myofibroblasts, correlating well with the number of *α*SMA‐positive cells, as shown in Figure 5B. As fibril diameter increases in the fibrotic tissues, we also quantified the mean fibril diameter of the decellularized matrices. We found that collagen fibril diameter remained similar in all matrix conditions, despite cell‐mediated changes in mean pore diameter (Figure 6C). We previously showed that fibril diameter increases only in the presence of M2a macrophages in macrophage–fibroblast cocultures.^[^ ^9^ ^]^ In addition to the mean pore and fibril diameter, we analyzed the matrix elasticity using a contactless rheometer. To better compare the change in matrix elasticity, the data were normalized to the elasticity of cell‐free matrices. As shown in Figure 6D, matrices of myofibroblasts and fibroblasts cultured in aligned matrices showed significantly higher changes in matrix elastic modulus when compared to fibroblasts cultured in both random matrices (0° and 0° + EDC). It appears that the aligned matrices (30° only) showed slightly higher elastic modulus than matrices from myofibroblasts with similar topological properties (15°); however, with no significant differences. These data highlight the acceleration of ECM stiffening in fibrotic tissue.

As matrix stiffening could be modulated by physical changes in the ECM or excessive production of ECM components, we studied differences in the production of matrix components using data obtained from RNASeq. As shown in Figure 6E, distinct expressions of matrix components in all matrix conditions are illustrated. We showed that stiffening of the random matrix (0° + EDC) significantly reduced the expression of COL6A1 and COL6A2, while it enhanced the expression of HYAL3 when compared to the more compliant random matrices (0°). Collagen alignment triggered the expression of COL4A1, COL4A2, COL7A1, COL8A2, FN1, and HAS2 but a reduction in HYAL3 when compared to both random matrices (0° and 0° EDC). Interestingly, myofibroblasts expressed more COL1A1, COL1A2, ELN, and LOX but showed lower expression of DCN, HAS3, HYAL2, LAMB1, LAMB2, NID1, and NID2 than cells cultured on aligned matrices (30°). Although cells cultured on aligned matrices possessed characteristics of a myofibroblast phenotype, the expression of matrix components appears to be dependent on fibril orientation.

In sum, our data support that the fibroblasts differentiated through aligned matrices possess a similar degree of matrix remodeling capabilities as myofibroblasts activated through TGF‐*β*1. Interestingly, matrix stiffness and orientation appear to regulate a distinct matrix component production regime by fibroblasts. These results suggest how specific microenvironments with specific ECM compositions and microarchitecture are made to assist the progression of disease, as well as maintain physiologically relevant conditions. For example, a gradual increase in collagen alignment might contribute to accelerating the tissue fibrotic condition by increasing matrix remodeling through cell–matrix interactions via enhanced expression of fibronectin, as previously reported.^[^ ^13^, ^40^ ^]^

### 2.6 Cell Contractility Mediates Fibroblast Differentiation in Aligned Collagen Matrices

**Figure 7:**
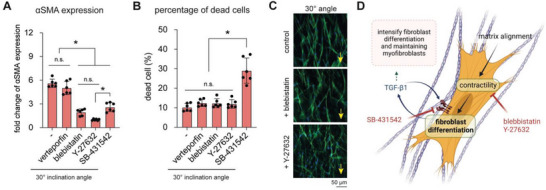
Inhibition of YAP, cecontractility, and TGF‐*β*1 signaling of fibroblasts in matrices with aligned collagen fibrils. Fibroblasts were cultured on matrix with aligned collagen fibrils (30° condition) in the presence of verteporfin, blebbistatin, Y‐27632, and SB‐43154 for 3 days. Cells were analyzed regarding A) *α*SMA expression and B) percentage of dead cells. For *α*SMA expression, data were normalized to fibroblasts. * indicates a significance level of *p* < 0.05 using the Mann–Whitney test. Experiments were performed in six replicates. C) Representative images of the cell orientation of cells cultured on matrix with aligned collagen fibrils (30° condition) with and without treatment with blebbistatin and Y‐27632. Cells were stained with Hoechst‐33421 (nucleus; blue) and phalloidin (actin; green) (scale bar = 50 µm). The arrow represents the direction of alignment. D) Proposed mechanotransduction mechanism by which collagen fibril alignment triggers fibroblast differentiation.

Matrix alignment appears to affect fibroblast behavior in different aspects. In an attempt to understand the underlying signaling pathway related to fibroblast differentiation in aligned collagen matrices (30°), we treated fibroblasts with chemical inhibitors, namely, verteporfin (yes‐associated protein (YAP) inhibitor), blebbistatin (myosin inhibitor), Y‐27632 (Rho kinase (ROCK) inhibitor), and SB‐431542 (TGF‐*β*1 receptor kinase inhibitor). Fibroblast differentiation was investigated by means of gene expression of *α*SMA using RT‐qPCR, as we have evidently shown it to be well correlated with the protein level of *α*SMA (Figure 5B,C) and as published elsewhere.^[^ ^9^, ^41^ ^]^ As shown in **Figure** **7**A, *α*SMA gene expression was significantly reduced when treated with blebbistatin, Y‐27632, and SB‐431542 but not with verteporfin. As has been reported, removing TGF‐*β*1 or reducing TGF‐*β*1 receptor activity could trigger apoptosis in myofibroblasts.^[^ ^41^, ^45^, ^48^ ^]^ To address this, we performed a live/dead assay and quantified the results via flow cytometry to check the viability of cells treated with different inhibitors. As shown in Figure 7B, only cells treated with SB‐431542 demonstrated a significant increase in cell death, which is in line with other reports^[^ ^49^, ^50^ ^]^ as TGF‐*β*1 is essential in maintaining myofibroblasts. This result also suggests that the reduction in *α*SMA by treatment with SB‐431542 could be caused by cell death, as previously reported.^[^ ^49^ ^]^ On the other hand, blebbistatin and Y‐27632 reduced *α*SMA gene expression without harming cells, suggesting that cell contractility might be associated with fibroblast differentiation in aligned matrices. In general, cell contractility is upregulated when the small GTPase RhoA activates its downstream effector ROCK. ROCK further upregulates phosphorylation of the myosin light chain. Therefore, inhibiting both myosin and ROCK downregulates cell contractility and can result in diffuse integrin distribution and a lack of focal adhesion in cells.^[^ ^51^ ^]^ Interestingly, treatment with both cell contractility inhibitors did not affect cell orientation in the direction of matrix alignment (Figure 7C), only *α*SMA expression. In line with our findings, cell contractility has been demonstrated to mechanically trigger fibroblast differentiation.^[^ ^13^, ^52^, ^53^, ^54^ ^]^ In addition, loss of myosin II has been found to inhibit fibroblast differentiation.^[^ ^55^ ^]^ Furthermore, pharmacological targeting of mechanical contraction in myofibroblasts leads to a reduction in the progression of fibrosis in a mouse model.^[^ ^56^ ^]^ In addition, other selective ROCK inhibitors can also reduce fibroblast differentiation and matrix remodeling in vitro.^[^ ^57^ ^]^ All these reports supported our finding that cell contractility might be a key player in fibroblast differentiation and could be a potential regulator in the mechanosensing of fibroblasts in response to matrix alignment.

In summary, we found that inhibiting cell contractility‐associated pathways, both through myosin or ROCK, could reduce fibroblast differentiation in aligned collagen, whereas inhibition of YAP signaling minimally affected differentiation. Inhibition of TGF‐*β*1‐associated signaling using SB‐431542 led to a reduction in *α*SMA expression due to cell death. The inhibition of cell contractility did not change the directional cell orientation in the direction of matrix alignment. A schematic illustration of the proposed mechanism of how matrix alignment triggered fibroblast differentiation is depicted in Figure 7D.

## 3 General Discussion and Conclusion

Collagen fibril alignment represents a key characteristic of fibrotic tissue.^[^ ^1^, ^2^, ^3^ ^]^ Recapitulating such microenvironments biomimetically in vitro will pave the way for understanding molecular mechanisms, better disease modeling, and finding novel therapeutic approaches for different pathologies. It has been shown that in vivo fibrosis models have been limited due to species differences between animal and human fibrosis mechanisms, which complicate the interpretation of findings for translation purposes.^[^ ^58^, ^59^, ^60^ ^]^ In addition, the complexity and heterogeneity of the tissue, including biophysical features and compositions, are distinct between different disease stages and can instruct different cell functions. Establishing a well‐defined model in which both biophysical and biochemical parameters can be fine‐tuned will provide an in‐depth understanding of how specific cues regulate distinct cell functions. As exemplified in this work, we established a simple, robust, and noninvasive approach to fine‐tune the fibril alignment degree of 3D collagen matrices. In addition, this approach allows for the reconstitution of 3D aligned matrices regardless of the concentration of collagen monomers (as shown in Figure S5, Supporting Information) and their source (as demonstrated in Figure S6, Supporting Information), while also providing the ability to modify fibril characteristics (as illustrated in Figure S7, Supporting Information). It also supports the 3R principle (replacement, reduction, and refinement) of animal usage in research. Using our established model, it could be shown that collagen fibril alignment can mechanically trigger fibroblast differentiation, which has been confirmed using functional transcriptome analysis and immunostaining of myofibroblast markers, as well as assessment of matrix remodeling capabilities. Fibroblast differentiation could not be observed in crosslinked random matrices, which exhibit higher bulk matrix stiffness than aligned matrices. Previous reports have indicated that the crosslinking of collagen using EDC could have an impact on the binding sites of integrins on cells, and in turn, affect cellular contractility and the differentiation of fibroblasts.^[^ ^61^ ^]^ In our studies, with or without EDC crosslinking, based on 𝛼SMA expression and cell proliferation, there were no differences in cellular functions of fibroblasts, but less matrix remodeling which reflected reduced cell contractility. This finding suggests that the alignment of collagen fibrils, rather than the overall stiffness of the matrix, triggers fibroblast differentiation. However, it remains unclear whether an increase in collagen mechanics within aligned collagen matrices would also facilitate the progression of fibroblast behavior. Our data also manifest that fibroblast differentiation in aligned matrices appears to be cell contractility dependent, which has been shown using inhibition of myosin II and ROCK. However, blocking cell contractility does not affect the cell orientation of fibroblasts, which might lead to fibroblast differentiation again after removing the inhibitor. In addition, we demonstrated that matrix organization and stiffness can trigger distinct expression of cytokines and matrix components in fibroblasts, creating specific cellular microenvironments. In summary, there is a contractility‐dependent fibrotic response to aligned collagen as seen through hallmark *α*SMA protein expression, characteristic cell function depicted by matrix remodeling, and transcriptomic analyses. This highlights the importance of physical cues in the regulation of cell functions, especially in the fibrosis and cancer microenvironment, where collagen alignment is mainly presented. It is important to note that collagen fibril alignment is not the only biophysical cue present in fibrotic tissues. Studies have shown that an increase in collagen fibril thickness can also trigger fibroblast differentiation, also via contractility.^[^ ^13^ ^]^ This highlights the importance of fibroblast cell contractility as a key mechanical cue in fibrotic formation and suggests that its associated pathways could be targeted for therapeutic interventions. However, it is still unclear to what extent collagen fibril thickness and alignment jointly modulate this process. Therefore, future research should aim to develop well‐defined biomimetic models that enable independent fine‐tuning of both biophysical parameters, in order to systematically investigate fibroblast behavior and anti‐fibrotic drugs. Overall, our established 3D aligned collagen matrices can be further extended for the in vitro engineering of cardiac and skeletal tissues, which can be used as a platform for disease modeling of these respective tissue systems, alongside fibrosis and cancer microenvironment studies.

## 4 Experimental Section

### Reconstitution of Collagen Matrices With and Without Fibril Alignment

Collagen solution was prepared according to the authors’ established protocol.^[^ ^62^ ^]^ Briefly, rat tail type I collagen (Advanced Biomatrix, Carlsbad, USA) was mixed with 0.1% acetic acid (Sigma–Aldrich, Germany) and 250 mm phosphate buffer at pH 7.5 (Sigma–Aldrich, Darmstadt, Germany) to achieve a collagen concentration of 2 mg mL^−1^.

For reconstitution of collagen alignment using inclined surfaces, a prepared collagen solution was placed onto a glutaraldehyde‐coated coverslip (13 mm in diameter; VWR, Darmstadt, Germany). Thereafter, the coverslip was transferred onto a planar surface (0°) or onto 3D printed inclined surfaces with angles of 7.5°, 15°, 22.5°, and 30°. The design of 3D printed inclined surfaces is illustrated in Figure 1A. Collagen fibrillogenesis was initiated at 37°C, 5% CO_2_, and 95% humidity. Reconstituted 3D collagen matrices were washed three times with phosphate buffer saline (PBS; Sigma–Aldrich, Darmstadt, Germany) and kept in PBS prior to performing further experiments.

For crosslinked matrices, reconstituted 3D matrices were treated with 20 mm 1‐ethyl‐3‐(3‐dimethyl aminopropyl)‐carbodiimide (EDC; Sigma–Aldrich, Darmstadt, Germany) prepared in 2‐(*N*‐morpholino) ethanesulfonic acid buffer (MES buffer, Sigma–Aldrich, Darmstadt, Germany) at 0.1 m and pH 5, as previously published.^[^ ^35^ ^]^ Matrices were incubated with crosslinking solution for 2 h at room temperature. Subsequently after chemical crosslinking, matrices were rinsed 5× with PBS (Sigma–Aldrich, Darmstadt, Germany) and equilibrated at these neutral conditions.

### Characterization of Topological and Mechanical Properties of 3D Collagen Matrices

Cell‐free 3D collagen matrices were analyzed to assess their topological and mechanical properties, as previously reported. Briefly, for topological analysis, collagen matrices were stained with 50 µm 5‐(and‐6)‐carboxytetramethylrhodamine succinimidyl ester (TAMRA‐SE, Sigma–Aldrich, Darmstadt, Germany) for 2 h at room temperature, as previously published.^[^ ^63^ ^]^ Matrices were visualized using a confocal laser scanning microscope (SP8; Leica, Wetzlar, Germany) with a 40× objective (NA 1.3). Acquired images were 1024 × 1024 pixels in resolution (*xyz*‐voxel size: 0.13 × 0.13 × 5 µm) and with a vertical stack size of 21 images (the total *z*‐stack was equivalent to 100 µm). The cLSM stacked images were analyzed in terms of mean pore and fibril diameter using a home‐built MATLAB script (MATLAB 2022a; MathWorks, Natick, USA), as described elsewhere.^[^ ^63^ ^]^ The distribution of fibril orientation and the coherence index (CI) index were quantified using obtained images using the OrientationJ plug‐in^[^ ^64^ ^]^ of ImageJ (NIH, Bethesda, USA). The CI was in the range between 0 and 1, where 0 corresponds to randomly oriented fibrils, and 1 corresponds to perfect alignment. The coherency was proven for unbiased characterization of fibril alignment when compared to other methods.^[^ ^32^ ^]^ The quantification was performed at four randomly selected positions of each sample from four independent samples.

The mechanical properties of cell‐free reconstituted collagen matrices were analyzed nondestructively via rheological measurement using ElastoSens Bio 2 (Rheolution, Montreal, Canada), as previously published.^[^ ^15^ ^]^ Briefly, prepared collagen solution was placed into the holder (Rheolution, Montreal, Canada) and polymerized onto a planar surface (0°) or 3D printed inclined surfaces with angles of 15° and 30°. For EDC crosslinked matrices, crosslinking solution was added to the reconstituted matrices, as described above in the collagen reconstitution section. Prior to assessment of mechanical properties, all matrices were washed 5× with PBS (Sigma–Aldrich, Darmstadt, Germany) and kept in a hydrated condition. For mechanical characterization of cell culture samples, matrices were decellularized by osmotic shock through incubation with distilled water for 1 h prior measurement, as previously reported.^[^ ^65^ ^]^ The mechanical characterization was performed at least in triplicate.

### Cell Culture

Primary human dermal fibroblasts (ATCC, Manassas, USA) were maintained in Dulbecco's modified Eagle medium (DMEM) supplemented with 10% fetal bovine serum and 1% penicillin–streptomycin. All cell culture materials were purchased from Thermo Fisher Scientific, Dreieich, Germany.

For cell culture, 3 × 10^4^ fibroblasts were seeded onto reconstituted 3D collagen matrices. Cells were cultured for 3 days under standard cell culture conditions (37 °C with 5% CO_2_ and 95% humidity). For differentiation of fibroblasts into myofibroblasts as a control, cells were cultured in DMEM supplemented with 10 ng mL^−1^ TGF‐*β*1 (Biolegend, San Diego, USA) for 3 days under standard cell culture conditions, as previously described.^[^ ^40^ ^]^

### Imaging‐Based Quantification of Cell Orientation

Cells were fixed with 4% paraformaldehyde (Biolegend, San Diego, USA) for 10 min at room temperature, followed by permeabilization using 0.1% Triton X100 (Merck KGaA, Darmstadt, Germany) for 10 min at room temperature. Afterward, cells were stained 24 h with Hoechst‐33420 for the cell nucleus (dilution 1:10 000 in PBS; Thermo Fisher Scientific, Dreieich, Germany) and phalloidin conjugated with Alexa Fluor 488 for the actin cytoskeleton (dilution 1:250 in PBS; Thermo Fisher Scientific, Dreieich, Germany). Images were gathered by epifluorescence microscopy (Leica, Wetzlar, Germany) using a 20× LD objective (NA 1.3; Leica, Wetzlar, Germany). Cell orientation was analyzed using the OrientationJ plug‐in^[^ ^64^ ^]^ of ImageJ (NIH, Bethesda, USA), as validated by Xu et al.^[^ ^66^ ^]^ The cell orientation index was in the range between 0 and 1, where 0 corresponds to randomly oriented cells, and 1 corresponds to a perfectly oriented cell. The quantification was performed at four randomly selected positions of each sample from four independent samples.

### RNA Sequencing and Analysis

For RNA isolation, TRIzol (Invitrogen, California, US) was used to extract the total RNA, followed by a purification step using the RNeasy mini kit (Qiagen, Hilden, Germany) as described by the manufacturer's protocol. RNA quantity and quality were quantified using a Nanodrop (Thermo Fisher Scientific, Dreieich, Germany) and a Qi RNA kit (Thermo Fisher Scientific, Dreieich, Germany). Samples were prepared with an NEB Ultra II RNA kit (New England Biolabs, Ipswich, MA, USA) according to the protocol instructions using the NEBNext Poly(A) mRNA Magnetic Isolation module (New England Biolabs, Ipswich, MA, USA), and uniquely dual indexed. The resulting library concentration, size distribution, and quality were assessed on a Qubit 4 fluorometer (Thermo Fisher Scientific, Inc., Dreieich, Germany) with a dsDNA high sensitivity kit (Invitrogen, Carlsbad, CA, USA) and on a 4200 TapeStation using a High Sensitivity D5000 kit (Agilent, Santa Clara, CA, USA). Based on these results, libraries were normalized according to their molarity and pooled and then quantified with a library quantification kit for Illumina platforms (Roche, Basel, Switzerland) on a StepOnePlus qPCR machine (Thermo Fisher Scientific, Dreieich, Germany). Finally, pooled libraries were loaded at 350 pm with 1% PhiX on S2 FlowCell and paired end sequenced (2 × 150 bp) on a NovaSeq 6000 next generation sequencer (Illumina, San Diego, USA). RNA‐Seq was performed in triplicate. The RNA‐Seq data processing procedure is provided as Supporting Information.

RNA‐Seq data were merged using the NASQAR toolbox^[^ ^67^ ^]^ (publicly accessible at http://nasqar.abudhabi.nyu.edu/), and the analysis was performed using iDEP 1.0^[^ ^68^ ^]^ (http://bioinformatics.sdstate.edu/idep/; publicly accessible by South Dakota State University). For the analysis of differentially expressed genes (DEGs), DEGs were analyzed with FDR cutoff ≤ 0.05 and FC ≥ 2.0 using DESeq2.^[^ ^69^ ^]^ Biological pathways were predicted using generally applicable gene‐set enrichment for pathway analysis (GAGE).^[^ ^70^ ^]^

### Quantitative Gene Expression Analysis

Gene expression analysis for fibroblast differentiation was performed using an established protocol, as published.^[^ ^71^ ^]^ Briefly, TRIzol (Thermo Fisher Scientific, Dreieich, Germany) was used to extract total RNA. The obtained RNA was converted into complementary DNA (cDNA) using a high‐capacity cDNA reverse transcription kit (Applied Biosystems, Waltham, USA). The concentration and the ratio of absorbance at 260 and 280 nm of cDNA were quantified using a Nanodrop (Thermo Fisher Scientific, Dreieich, Germany) prior to performing gene expression analysis. Ribosomal protein S26 (RPS26) was used as a reference gene. The primers were synthesized by Bioneer (Daejeon, Republic of Korea). The primer sequences are listed in Table S1, Supporting Information. qPCR was performed using SYBR Green PCR Master Mix (Applied Biosystems, Waltham, USA). The qPCR procedure was set as follows: denaturation for 5 min at 95 °C; 45 cycles of denaturation (95 °C, 15 s), annealing under primer‐specific conditions (30 s), and target gene‐specific extension (30 s at 72 °C). The fluorescence signal was measured for 20 s at 72 °C. To confirm the specificity of the PCR products, melting curve analysis was performed at the end of each run. Experiments were performed in four replicates.

### *α*SMA Staining and Image Analysis

Cells were briefly fixed with 4% paraformaldehyde (Biolegend, San Diego, USA) for 10 min at room temperature, followed by permeabilization using 0.1% Triton X100 (Merck KGaA, Darmstadt, Germany) for 10 min at room temperature. Afterward, cells were stained for 24 h with Hoechst‐33420 for the cell nucleus (dilution 1:10 000 in PBS; Thermo Fisher Scientific, Dreieich, Germany) and phalloidin conjugated with Alexa Fluor 549 for the actin cytoskeleton (dilution 1:250 in PBS; Thermo Fisher Scientific, Dreieich, Germany). For additional staining of alpha‐smooth muscle actin (*α*SMA), cells were blocked with 1% bovine serum albumin for 1 h at room temperature, incubated with mouse anti‐human *α*SMA (dilution 1:250 in PBS; Biolegend, San Diego, USA) overnight at 4  °C, and incubated with goat anti‐mouse IgG conjugated with Alexa Fluor‐488 (dilution 1:250 in PBS; Thermo Fisher Scientific, Dreieich, Germany) for 2 h. Cells were washed three times with PBS after each step. Cell imaging was performed using an epi‐fluorescence microscope (DMi8 S; Leica, Wetzlar, Germany) using a 20× LD objective (NA 1.3; Leica, Wetzlar, Germany). The percentage of *α*SMA‐positive cells was determined by manual counting of cells positive for *α*SMA. The quantification was performed at four randomly selected positions of each sample from four independent samples.

### Quantitative Analysis of Cytokine Secretion

To analyze cytokines secreted by cells, cell culture supernatants were collected after 3 days of culture. Bead‐based multiplex immunoassay for CCL2, CXCL8, IL6, TGF‐*β*1, and VEGF (Biolegend, San Diego, USA) was utilized to quantify cytokines following instructions by the manufacturer. Samples were analyzed using an Attune NxT Flow Cytometer equipped with an autosampler (Thermo Fisher Scientific, Dreieich, Germany). Data analysis was performed by applying a five‐parameter curve fitting algorithm using LEGENDplex data analysis software (Biolegend, San Diego, USA). Experiments were performed in four replicates.

### Treatment With Inhibitors

Fibroblasts cultured onto matrix reconstituted on inclined surfaces with an angle of 30° were treated with 1 µm verteporfin (YAP inhibitor; Sigma–Aldrich, Darmstadt, Germany), 10 µm blebbistatin (myosin inhibitor; Tocris, Bristol, United Kingdom), 20 µm Y‐27632 (Rho‐associated kinase (ROCK) inhibitor; Tocris, Bristol, United Kingdom), and 2 µm SB‐431542 (TGF‐*β*1 receptor kinase inhibitor; Sigma–Aldrich, Darmstadt, Germany) for 3 days. After 3 days of treatment, RNA was extracted for the study of *α*SMA expression using qPCR, as stated in the quantitative gene analysis section. Experiments were performed in six replicates.

### Quantitative Analysis of Cell Number and Dead Cells

Collagen matrices were digested using 2 mg mL^−1^ type IV collagenase (Thermo Fisher Scientific, Dreieich, Germany) for 15 min under standard cell culture conditions. Afterward, cells were stained with DRAQ7 (dead cell staining dye; Biolegend, San Diego, USA) for 10 min on ice. Cells were analyzed using an Attune NxT Flow Cytometer equipped with an autosampler (Thermo Fisher Scientific, Dreieich, Germany). To analyze cell proliferation, the cell count obtained from the flow cytometer was normalized to the number of cells cultured on a random matrix (0°). The percentage of dead cells was quantified by counting the number of cells that were positive for DRAQ7. At least 20 000 cells were analyzed per condition and replicate. Experiments were performed in six independent replicates.

### Statistical Analysis

Experiments were performed in at least four replicates unless otherwise stated. Error bars indicate standard deviation (SD). Levels of statistical significance were determined by a Mann–Whitney test using GraphPad Prism 9 (GraphPad Software, San Diego, USA). The significance level was set at *p*  <  0.05.

## Conflict of Interest

The approach to fabricate aligned collagen is intellectually protected. U.S. Patent Application No. 63/486,676 Titled "Non‐Invasive Tuning of Collagen Microarchitecture to Recapitulate the Progression of Tissue Fibrosis"

## Supporting information

Supporting Information

## Data Availability

The data that support the findings of this study are available from the corresponding author upon reasonable request.
