# Supplementary material for: Collagen Fibril Orientation Instructs Fibroblast Differentiation Via Cell Contractility
Source: Adv Sci (Weinh). 2023 May 30;10(22):2301353. doi: 10.1002/advs.202301353 (PMC10401101; doi:10.1002/advs.202301353)
Supplement: Supplementary file 1 — Supporting Information [file ADVS-10-2301353-s001.pdf]

ADVANCED SCIENCE
Open Access

Supporting Information

for *Adv. Sci.*, DOI 10.1002/advs.202301353

Collagen Fibril Orientation Instructs Fibroblast Differentiation Via Cell Contractility

*Jiranuwat Sapudom, Shaza Karaman, Brian Chesney Quartey, Walaa Kamal Eldin Mohamed, Nick Mahtani, Anna Garcia-Sabaté and Jeremy Teo**

Supporting Information

Collagen fibril orientation instructs fibroblast differentiation via cell contractility

Jiranuwat Sapudom[1], Shaza Karaman[1], Brian Chesney Quartey[1], Walaa Kamal Eldin Mohamed[1],Nick Mahtani[2], Anna Garcia-Sabaté[1],and Jeremy Teo[1,3,]*

Processing of RNA Sequencing data

Raw FASTQ sequenced reads were first assessed for quality using FastQC v0.11.5 (available online at http://www.bioinformatics.babraham.ac.uk/projects/fastqc/)[1]. The reads were then passed through Trimmomatic v0.36[2] for quality trimming and adapter sequence removal with the following parameters (*ILLUMINACLIP: trimmomatic_adapter.fa:2:30:10 TRAILING:3 LEADING:3 SLIDINGWINDOW:4:15 MINLEN:36*). The surviving trimmed read pairs were then processed with Fastp[3] to remove poly-G tails and Novaseq/Nextseq-specific artifacts. Following quality trimming, the reads were assessed again using FastQC. After QC and QT, the reads were aligned to the human reference genome GRCh38.p4 using HISAT2[4] with the default parameters and by providing the *–dta* flag. The resulting SAM alignments were then converted to BAM format and coordinate sorted using SAMtools v1.3.1[5]. The sorted alignment files were then passed through HTSeq-count v0.6.1p1[6] using the following options (*-s no -t exon -I gene_id*) for raw count generation. Concurrently, the sorted alignments were processed through Stringtie v1.3.0[7] for transcriptome quantification. Briefly, the process was stringtie -> stringtie merge (to create a merged transcriptome GTF file of all the samples) -> stringtie (this time using the GTF generated by the previous merging step). Finally, Qualimap v2.2.2[8] was used to generate RNA-Seq-specific QC metrics per sample.

References

[1] S. Andrews, **2010**.
[2] A. M. Bolger, M. Lohse, B. Usadel, *Bioinformatics* **2014**, *30*, 2114.
[3] S. Chen, Y. Zhou, Y. Chen, J. Gu, *Bioinformatics* **2018**, *34*, i884.
[4] D. Kim, B. Langmead, S. L. Salzberg, *Nat Methods* **2015**, *12*, 357.
[5] H. Li, B. Handsaker, A. Wysoker, T. Fennell, J. Ruan, N. Homer, G. Marth, G. Abecasis, R. Durbin, *Bioinformatics* **2009**, *25*, 2078.
[6] S. Anders, P. T. Pyl, W. Huber, *Bioinformatics* **2015**, *31*, 166.
[7] M. Pertea, D. Kim, G. M. Pertea, J. T. Leek, S. L. Salzberg, *Nat Protoc* **2016**, *11*, 1650.
[8] F. García-Alcalde, K. Okonechnikov, J. Carbonell, L. M. Cruz, S. Götz, S. Tarazona, J. Dopazo, T. F. Meyer, A. Conesa, *Bioinformatics* **2012**, *28*, 2678.

Table S1: Primer list

Gene	Primer	Sequence (5'→3')	Accession number
RPS26	forward	CAATGGTCGTGCCAAAAAG	NM_001029
	reverse	TTCACATACAGCTTGGGAAGC	
αSMA (ACTA2)	forward	AGACCCTGTTCCAGCCATC	NM_001141945.1
	reverse	TGCTAGGGCCGTGATCTC	

Table S2: Comparison of various techniques for reconstituting 3D collagen matrices with fibril alignment.

Method	Ease of fabrication	Adjustable alignment degree	Reproducibility	Throughput
Microfluidic devices	++	++	++	+
Paramagnetic microbeads	++	+	+	+
Cyclic mechanical stretching	++	+	++	+
Constrained cellular compaction	+	+	+	+
Microextrusion 3D printing	++	+	+++	+++
Inclined surface (this work)	+++	+++	+++	+++

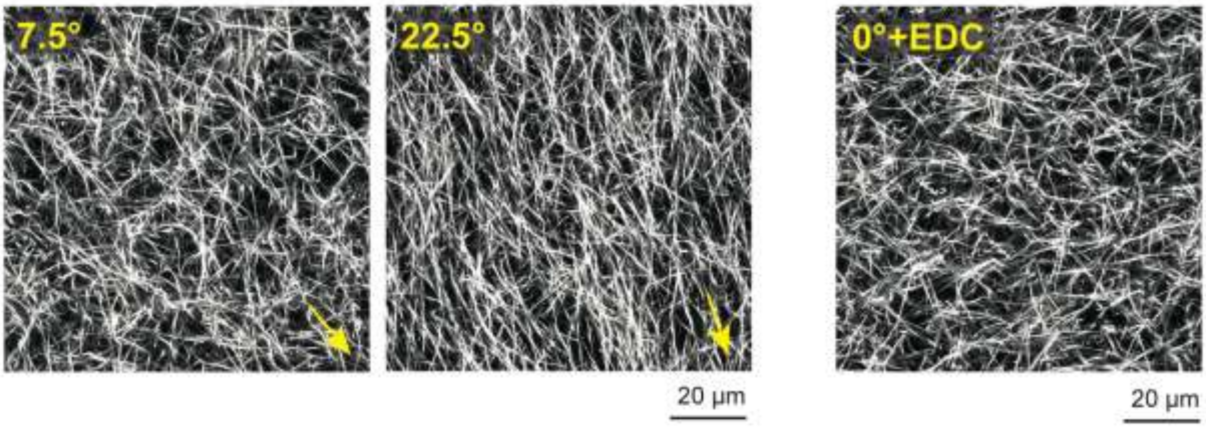

Figure S1: Microstructure of reconstituted matrices. Representative image of collagen matrices reconstituted on inclined surfaces with angles of 7.5°, 22.5° and 0° with postmodification with EDC crosslinker (scale bar=20 µm). Yellow arrows represent the direction of alignment.

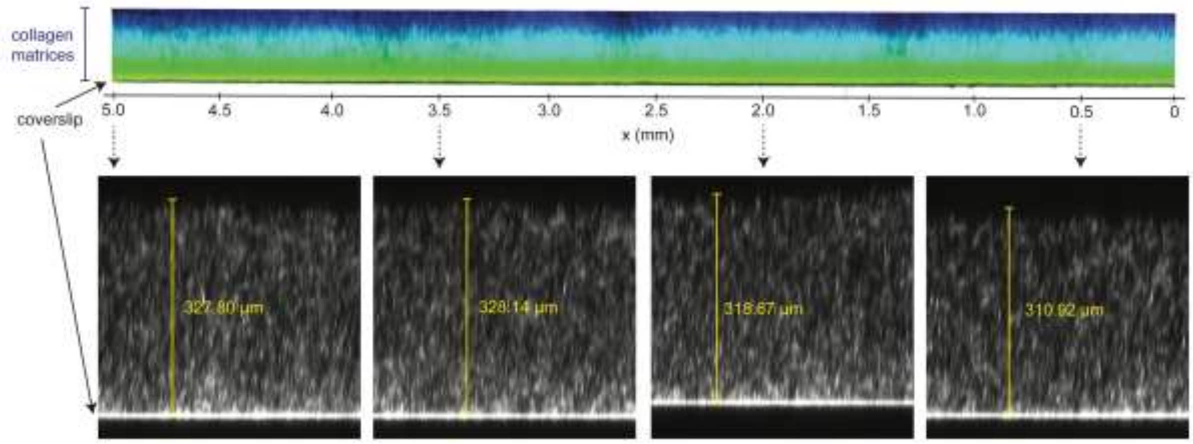

Figure S2: Thickness of collagen matrices reconstituted on inclined surface with an angle of 30°. Representative confocal image shows a cross-sectional view of a 5 mm xz-section of collagen matrices that were reconstituted on inclined surfaces with angles of 30°. Image was gathered using 10× objective (Leica, Wetzlar, Germany). Depth coding profile - color corresponds to the depth from the collagen surface, with blue being closest to the surface and red to the coverslip. In addition, zoomed-in images of four different sections were illustrated, and the collagen thickness was measured (indicated by yellow text and scale). The image demonstrates that the thickness of the collagen reconstituted on the inclined surface minimally changed with an incline of approximately 0.23°.

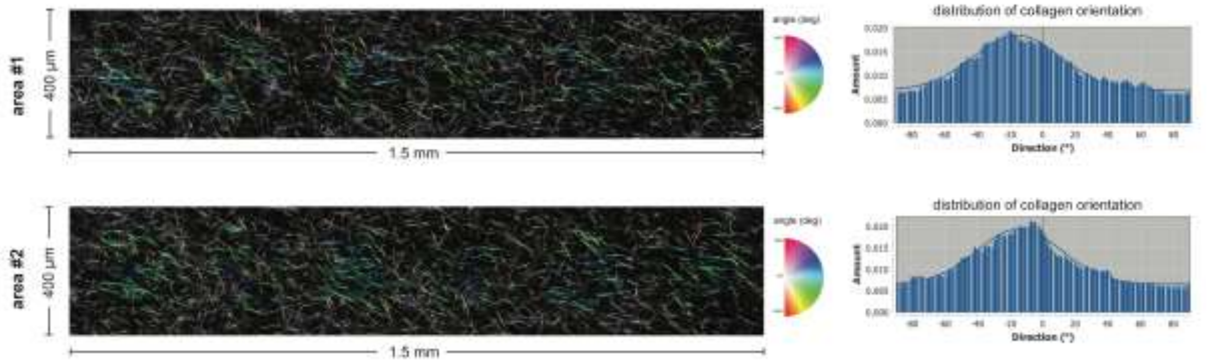

Figure S3: Long-range images of collagen matrices with aligned fibrils. Representative images of xy-sections were obtained from collagen matrices reconstituted on an inclined surface with an angle of 30°. Images from two positions were acquired using a 40× oil immersion objective (Leica, Wetzlar, Germany) and analyzed using the OrientationJ plug-in of Fiji. Colors in the images indicate fibril orientation, and the distribution of fibril direction for each position was also plotted. The images demonstrate that collagen alignment can be observed on a long-range scale of the matrix.

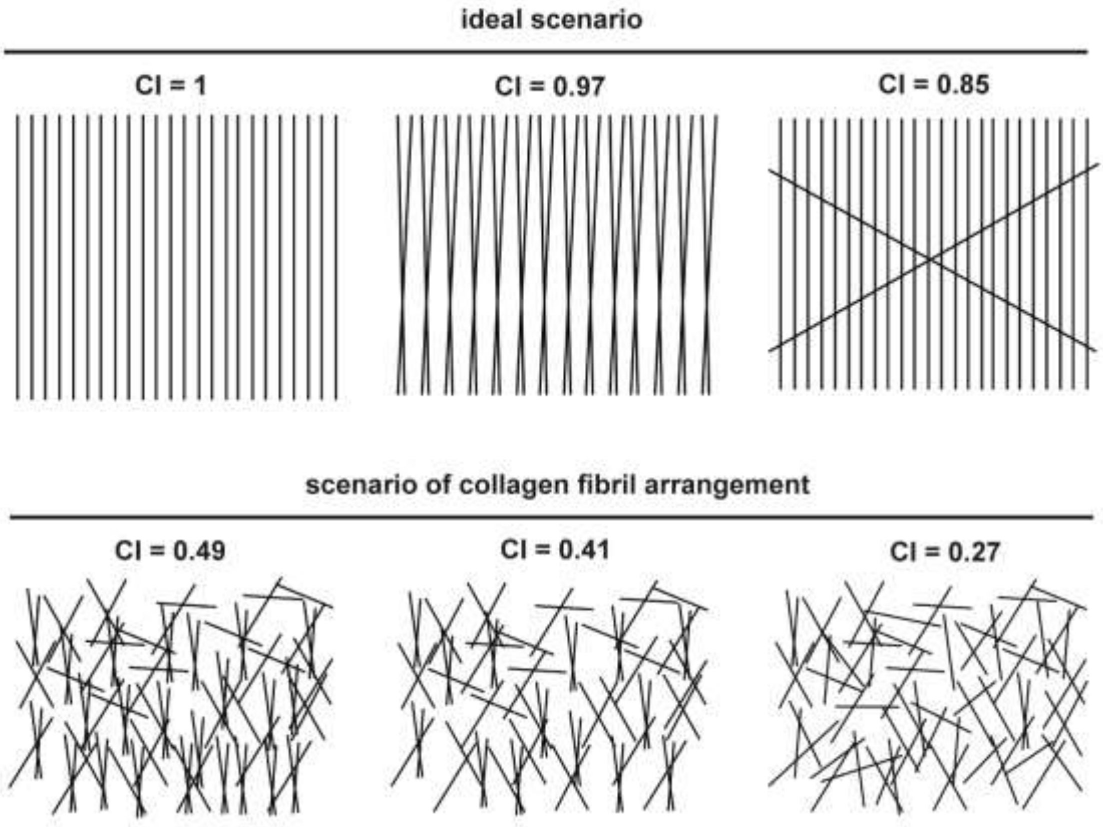

Figure S4: The coherence index of an aligned (ideal) scenario and a simulated collagen fibril arrangement. Different patterns were manually generated, and the coherence index (CI) was quantified for each pattern using the OrientationJ plug-in of Fiji software. The image highlights the difference in CI between a perfectly aligned (ideal) scenario and a simulated scenario of collagen fibril arrangement. It can be observed that, in the collagen fibril scenario, an increase in the number of aligned fibrils leads to a slight change in the CI value, from 0.41 to 0.49.

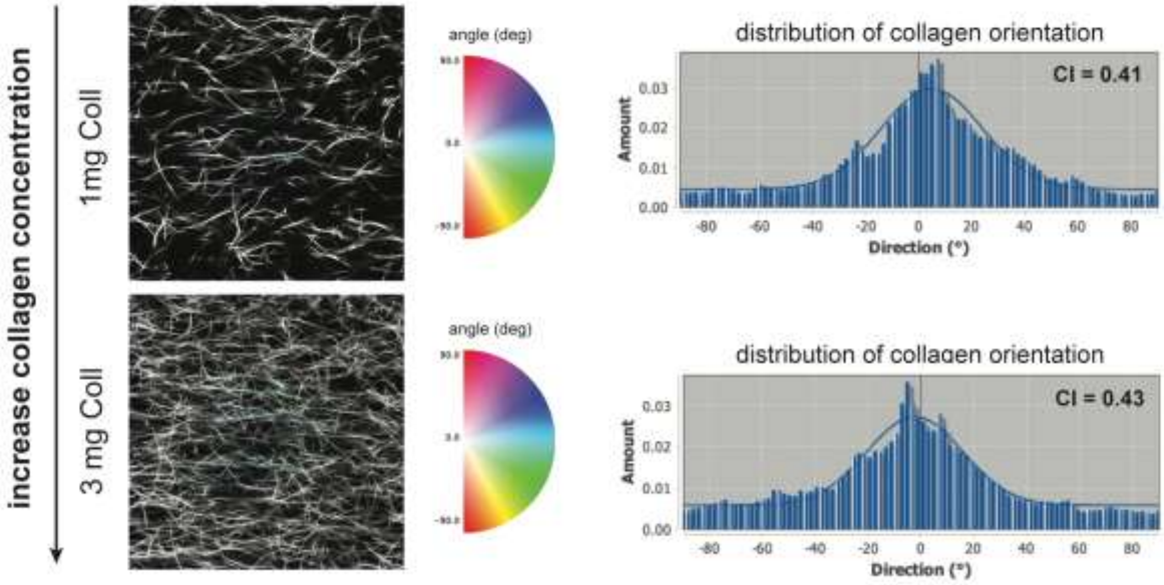

Figure S5: The reconstruction of aligned collagen matrices with different collagen concentrations. Representative images were obtained from collagen matrices reconstituted on an inclined surface with an angle of 30° at collagen concentrations of 1 mg/mL and 3 mg/mL. Images were analyzed using the OrientationJ plug-in of Fiji, and colors in the images indicate fibril orientation. The distribution of fibril direction for each position was also plotted. The result demonstrate that our developed method can be used to reconstitute collagen matrices with different types of collagen and concentrations, which enables us to mimic various physiological and pathological conditions.

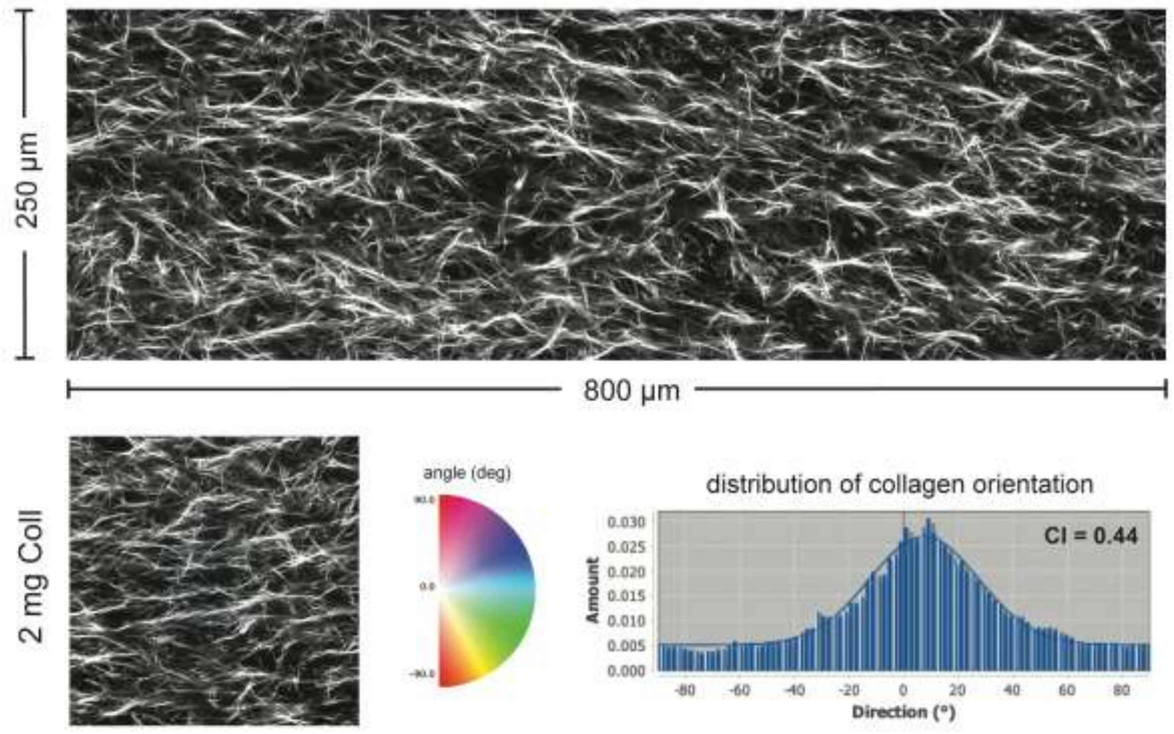

Figure S6: The reconstruction of aligned collagen matrices with type I bovine collagen. Representative images were obtained from collagen matrices reconstituted on an inclined surface with an angle of 30° at a concentration of 2 mg/mL with 250 mM phosphate buffer at pH 7.5 and pH 6.5, which is similar 9ot he type I rat tail collagen used in this study. Images were analyzed using the OrientationJ plug-in of Fiji, and colors in the images indicate fibril orientation. The distribution of fibril direction for each position was also plotted. The result demonstrate that our developed method can be used to reconstitute collagen matrices with other sources of type I collagen.

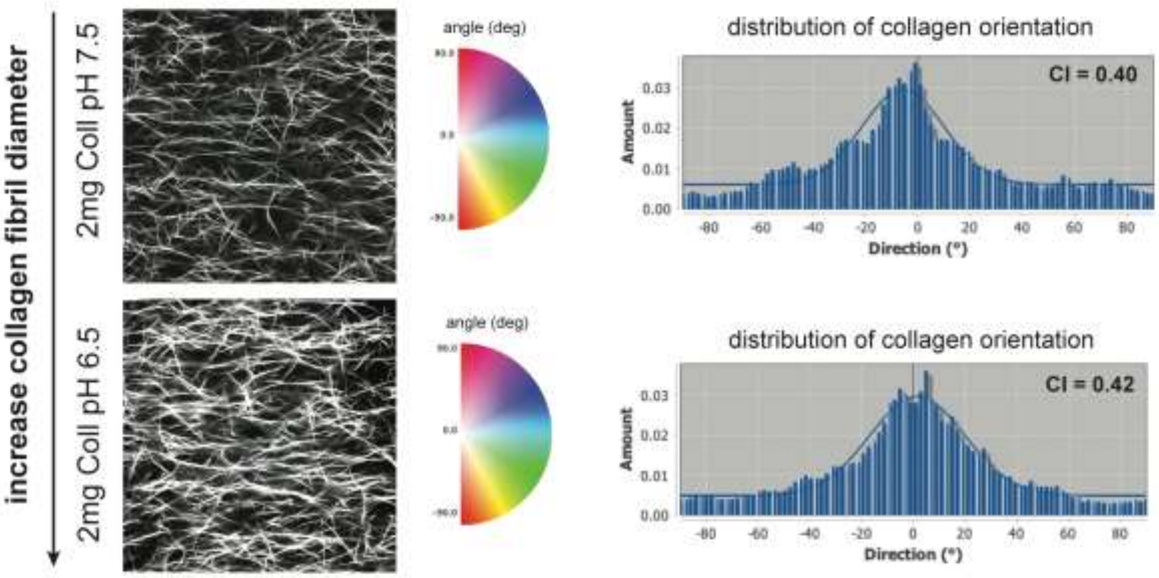

Figure S7: The reconstruction of aligned collagen matrices with different fibril thicknesses. Representative images were obtained from collagen matrices reconstituted on an inclined surface with an angle of 30° at a collagen concentration of 2 mg/mL 250 mM phosphate buffer at pH 7.5 and pH 6.5, as described by Sapudom et al. (2015) in Biomaterials. Images were analyzed using the OrientationJ plug-in of Fiji, and colors in the images indicate fibril orientation. The distribution of fibril direction for each position was also plotted. The result demonstrate that our developed method can be used to reconstitute collagen matrices with different types of collagen and concentrations, which enables us to mimic various physiological and pathological conditions.
